# Whole adult organism transcriptional profiling of acute metal exposures in male Zebrafish

**DOI:** 10.1186/2050-6511-15-15

**Published:** 2014-03-10

**Authors:** Naissan Hussainzada, John A Lewis, Christine E Baer, Danielle L Ippolito, David A Jackson, Jonathan D Stallings

**Affiliations:** 1 ORISE Postdoctoral Fellow, Ft. Detrick, Frederick, Maryland 21702, USA; 2 Biomarkers Program, US Army Center for Environmental Health Research, Fort Detrick, Frederick, Maryland 21702-5010, USA; 3 Pulmonary Health Program, US Army Center for Environmental Health Research, Fort Detrick, Frederick, Maryland 21702-5010, USA; 4 Excet Inc., Fort Detrick, Frederick, Maryland 21702-5010, USA; 5 ORISE, Fort Detrick, Frederick, Maryland 21702-5010, USA; 6 Environmental Health Program, US Army Center for Environmental Health Research, Fort Detrick, Frederick, Maryland, USA

**Keywords:** Metals, Toxicity mechanisms, Zebrafish, Whole organism, Nickel, Chromium, Cobalt, Toxicogenomics

## Abstract

**Background:**

A convergence of technological breakthroughs in the past decade has facilitated the development of rapid screening tools for biomarkers of toxicant exposure and effect. Platforms using the whole adult organism to evaluate the genome-wide response to toxicants are especially attractive. Recent work demonstrates the feasibility of this approach in vertebrates using the experimentally robust zebrafish model. In the present study, we evaluated gene expression changes in whole adult male zebrafish following an acute 24 hr high dose exposure to three metals with known human health risks. Male adult zebrafish were exposed to nickel chloride, cobalt chloride or sodium dichromate concentrations corresponding to their respective 96 hr LC_20_, LC_40_ and LC_60_. Histopathology was performed on a subset of metal-exposed zebrafish to phenotypically anchor transcriptional changes associated with each metal.

**Results:**

Comparative analysis identified subsets of differentially expressed transcripts both overlapping and unique to each metal. Application of gene ontology (GO) and transcription factor (TF) enrichment algorithms revealed a number of key biological processes perturbed by metal poisonings and the master transcriptional regulators mediating gene expression changes. Metal poisoning differentially activated biological processes associated with ribosome biogenesis, proteosomal degradation, and p53 signaling cascades, while repressing oxygen-generating pathways associated with amino acid and lipid metabolism. Despite appreciable effects on gene regulation, nickel poisoning did not induce any morphological alterations in male zebrafish organs and tissues. Histopathological effects of cobalt remained confined to the olfactory system, while chromium targeted the gills, pharynx, and intestinal mucosa. A number of enriched transcription factors mediated the observed gene response to metal poisoning, including known targets such as p53, HIF1α, and the *myc* oncogene, and novel regulatory factors such as XBP1, GATA6 and HNF3β.

**Conclusions:**

This work uses an experimentally innovative approach to capture global responses to metal poisoning and provides mechanistic insights into metal toxicity.

## Background

Toxicogenomics is a powerful tool for evaluating toxicity profiles of known and potentially hazardous compounds. The zebrafish, a classic model for developmental toxicity, has recently proven to be an effective model organism for chemical screening [1,2] and environmental sentinel applications, including sewage testing and chemical hazard detection [3-6]. The low husbandry costs, small size, ease of genetic manipulation, and wealth of genome database resources distinguish the zebrafish as a highly promising model organism for toxicological studies. Responses to toxic insults usually affect multiple organs and tissues, supporting a role for gene profiling in the whole animal to evaluate toxic responses. Although whole organism toxicogenomics has routinely been conducted in invertebrate models such as the worm *Caenorhabditis elegans* and the fly *Drosophila melanogaster*[7-9] and in ecoindicator species such as *Daphnia magna* and *Pimpephales promelas*[10,11], only one study to date has evaluated whole organism gene profiling in adult zebrafish [12]. In this study, robust expression signatures differentiated between potent aryl hydrocarbon and estrogen receptor agonists, and accurately identified target tissues. We hypothesized that gene profiling in whole adult zebrafish could also be used to infer toxic responses to hazardous chemicals.

Nickel, cobalt and chromium are environmentally ubiquitous metals with recognized human health hazards [13]. Recently, applications in mining, smelting, industry, medicine, and agriculture have increased the environmental distribution of these metals, elevating elevated exposure risk and incidences of occupational exposure. Nickel, cobalt, or chromium exposure can cause incapacitating acute toxicity and/or long-term damage (e.g., carcinogenesis) [14-16]. Primary mechanisms of metal toxicity include the production of free radicals which can trigger oxidative stress, induce mutagenesis by DNA-metal interactions, and impair protein function by covalently modifying proteins or competing with metal binding sites. Metal-derived reactive oxygen species (ROS) may perturb a number of tightly regulated cellular processes (e.g., cell growth and proliferation), activate transcription factors and genes, and trigger cellular adaptive programs including metal stress response, DNA repair mechanisms, and inflammation [17].

Numerous studies have examined gene responses to acute poisoning by nickel, cobalt, or chromium [18,19], but besides a few studies in invertebrates, most of these studies measure gene responses in isolated tissues or tissue-derived cell lines [8,19-23]. Although analyzing isolated tissues is the ideal approach to unambiguously identify gene changes in an organ of interest, it is experimentally impractical to microdissect and to analyze all potentially affected organs from zebrafish individually. Alternatively, a whole-organism approach with *post hoc* gene ontology enrichment analysis has the advantage of predicting biomolecular pathways linked to observed histopathologic endpoints for informing later organ-specific experiments. In this study, we used a whole-organism approach exposing adult male zebrafish to increasing concentrations of nickel chloride, cobalt chloride, and sodium dichromate, and evaluating whole genome transcriptional responses using DNA microarrays. We identified differentially regulated biological processes using gene ontology enrichment analysis in order to infer toxicity mechanisms [21]. We also identified transcription factors upstream of the differentially enriched genes which are predicted to directly activate or repress gene expression in order to characterize regulatory processes involved in metal toxicity. Histopathological changes in the whole organism were compared with gene changes. Overall, our study provides (i) insight into transcriptomic changes corresponding to toxic indicators of metal poisoning and (ii) an experimental evaluation of whole organism toxicogenomics in the zebrafish model.

## Methods

Research was conducted in compliance with the United States Animal Welfare Act, and other Federal statutes and regulations relating to animals and experiments involving animals and adheres to principles stated in the Guide for the Care and Use of Laboratory Animals (NRC 2011) in facilities that are fully accredited by the Association for the Assessment and Accreditation of Laboratory Animal Care, International. Approvals were granted for this study by USACEHR’s Institutional Animal Care and Use Committee.

### Water quality

All husbandry and aquatic exposures are performed using USACEHR’s well water which is processed to ensure proper conditioning of the water supply. The water is supplied from a mix of onsite ground water wells and from municipal tap (domestic) water. Domestic water is used to produce “RO permeate” which is later mixed with raw well water to produce water of appropriate hardness and alkalinity (150 – 210 mg/L as CaCO_3_; 110 – 180 mg/L as CaCO_3_ respectively). Domestic water is carbon filtered to remove chlorine levels (maintained below 0.1 mg/L), treated by a water softener, processed through reverse osmosis (RO) membranes then stored for distribution. Prior to use, the RO processed domestic water is blended with well water, filtered through a 10 μm particle filter, carbon filtered, then heated and aerated to near 100% saturation at 25 ± 1°C. This processed water is then passed through another 10 μm particle filter and UV sterilized prior to distribution throughout the facility. This water is continuously monitored to maintain the following ranges: pH = 6.5 – 8.5; alkalinity = 110 – 180 mg/L CaCO_3_; hardness = 150 – 210 mg/L as CaCO_3_; conductivity = 400 – 1000 mS/cm; total ammonia < 0.1 mg/L as NH_3_; dissolved oxygen (DO) = 80 – 100% saturation (6.8 – 8.5 mg/L at 25°C). Contaminant analysis is performed quarterly by our in-house analytical chemistry department as well as annually by an external, certified testing facility.

### Fish exposures

Exposures were conducted using USACEHR well water in 5-gallon glass aquaria adapted for flow-through use (60 mL/min; 5.4 turnovers/day) and maintained at 25°C with a 12 hr:12 hr (light:dark) photoperiod. During both acclimation and exposure periods, water quality for each tank is monitored daily (temperature, pH, alkalinity, hardness, DO, and conductivity (data not shown). We estimated the concentration of each metal necessary for 20% (LC_20_; low), 40% (LC_40_; mid) and 60% lethality (LC_60_; high) in 96 hr range-finding experiments. Exposures were conducted for 24 hr using control (no toxicant) plus the high, mid and low concentrations of each metal (Table 1). Our intent with this exposure paradigm was to evaluate levels of toxicant sufficient to induce a measurable intoxication response without producing lethality at 24 hours. Metal concentrations in the test tanks were verified by our analytical chemistry department before and after exposures.

**Table 1:** Nominal versus measured concentrations of metals

**Treatment**	**Nominal LC**_**20**_**(low)**	**Measured LC**_**20**_**(low)**	**Nominal LC**_**40**_**(mid)**	**Measured LC**_**40**_**(mid)**	**Nominal LC**_**60**_**(high)**	**Measured LC**_**60**_**(high)**
NiCl_2_	45	42.4	54	51.0	62	64.0
CoCl_2_	39	39.7	50	46.3	65	59.5
Na_2_Cr_2_O_7_	53	56.5	65	69.9	76	80.6

Nominal and actual concentrations (mg/L) of metals used in the definitive 24-hour zebrafish exposures as estimated from 96-hour range finding studies.

Only male zebrafish were included in the analysis because of concern that RNAs encoding vitellogenin and other liver-abundant egg proteins found in breeding females [24,25] might confound global gene expression studies. Therefore, we initially selected 25 adult (6–9 months) presumptive male zebrafish per condition to ensure that 20 male fish were available for subsequent microarray analysis and histopathology. During exposure, animals received a pre-measured quantity of food twice per day (1X flake food, 1X brine shrimp). After the exposure period, fish were euthanized by immersion in a lethal concentration (0.5 g/L, pH 7.2) of MS-222. Five fish per condition were immediately preserved in a modified Davidson’s solution for histological examination. For transcriptional analysis, the remaining 15 zebrafish were immersed whole in liquid nitrogen and stored at -80°C until RNA processing.

### Histopathology

Slides were prepared by Experimental Pathology Laboratories, Inc. (EPL, Inc., Sterling, VA). Briefly, the fish were initially preserved in modified Davidson’s solution, washed in 70% ethanol, and then transferred to 10% neutral buffered formalin for transport. Fish required additional decalcification prior to sectioning and were placed in Formical 2000® decalcification fluid for seven hours. Tails were removed from each fish, followed by processing and embedding in paraffin. Vertical longitudinal sections were obtained at five different levels: 1) left lateral, 2) left paramedian, 3) midline sagittal, 4) right paramedian, and 5) right lateral. Two serial sections were obtained at each level for a total of 10 sections per fish that were H&E stained. The following tissues were evaluated (if present) for each zebrafish: bone (vertebra), brain, corpuscle of Stannius, esophagus, eye, gallbladder, gills, heart, gonad (ovary), gonad (testis), hematopoietic tissue, interrenal tissue, intestine, kidney, liver, mesonephric duct, nares, pancreas, peripheral nerve, pineal organ, pituitary, pseudobranch, skeletal muscle, skin, spinal cord, spleen, stato-acoustic organ, swim bladder, thymus, thyroid, and ultimobranchial body. The following tissues occasionally were not present in the sections that were evaluated: corpuscle of Stannius, esophagus, gallbladder, interrenal tissue, mesonephric duct, pineal organ, pituitary, spleen, thymus, thyroid, and ultimobranchial body. Occasional absence of these tissues is a condition inherent in the sectioning method and did not appear to affect the overall evaluation of the histopathology data.

### Microarray analysis

#### RNA processing

Whole frozen fish were pulverized under liquid nitrogen using a SPEX 6750 freezer mill (SPEX Sample Prep, Metuchen, NJ). Total RNA was isolated from the pulverized material using Trizol® (Invitrogen, Carlsbad, CA) with an extra clarification centrifugation step to remove bone, scales, lipid, and other insoluble debris followed by column purification with RNeasy® Midi kits (Qiagen, GmbH, Germany) to remove residual salt and organic solvents. Total RNA quality and quantity were evaluated using an Agilent Bioanalyzer 2100 (Agilent, Santa Clara, CA) and verified using the NanoDrop ND-1000 Spectrophotometer (NanoDrop, Wilmington, DE). A portion of each total RNA preparation was reverse transcribed into cDNA using the Advantage® RT-for-PCR Kit (Clontech, Mountain View, CA) and screened against a primer panel designed to verify that RNA was isolated from male fish. Specifically, we measured levels of transcripts coding for vitellogenin 1 (vit1, expressed only in female liver, and glyceraldehyde 3-phosphate dehydrogenase (GAPDH), which was used as an internal control for normalizing the sample RNA and cDNA concentrations. Our initial PCR screen was critical as multiple RNA samples were pooled for microarray analysis (see below) and the presence of female RNA within the pool would complicate analysis.

#### Microarray hybridization

To maximize statistical power and minimize cost, we pooled equal amounts of total RNA from four or five fish within each exposure condition to create a biological replicate pool and hybridized each replicate pool to a separate microarray; generating four biological replicate pools per experimental condition for a total of 16 microarrays per toxicant screened (i.e. four control replicates, four low dose replicates, four mid dose replicates and four high dose replicates). Statistical modeling demonstrates that performing microarray analysis on four biological replicates comprised of RNA pooled from five samples approaches the statistical power attained by analyzing 20 individual samples [26]. Numerous theoretical discussions of the pooling procedure can be found in the literature [27-29]. Although pooling eliminates the ability to assess fish-to-fish variability in gene expression, it does provide a statistically powerful approach to identify clear toxicant responses, which is the main focus of the current work.

The microarrays used in this study were custom designed in-house using the eArray microarray design tool (https://earray.chem.agilent.com/earray/; Agilent Technologies, Inc.) and manufactured by Agilent. Each array contains 44,000 60-mer oligonucleotides representing 21,904 zebrafish gene targets derived from Ensembl build 46 (Zv7 genome build) and Vega build 26. Two probes were designed per transcript wherever possible; only 94 target transcripts have only one probe. Probes were designed using genes that are annotated, i.e., matched to named genes in the published databases, and represent good coverage of the whole zebrafish genome.

Microarrays were processed following Agilent’s One-Color Microarray-Based Gene Expression Analysis Protocol (Version 5.5, February, 2007) for processing 4 x 44 K microarray slides using an initial 1 μg pooled RNA input and an 18 hr overnight hybridization at 65°C. A final step for preventing ozone related degradation of signal using the Stabilization and Drying solution (Agilent Technologies, Inc.) was included after the required specificity washes prior to scanning the arrays. Microarray slides were scanned with a GenePix Autoloader 4200 AL scanner (Molecular Devices, Union City, CA) and raw images processed using GenePix Pro 6.0 (Molecular Devices). All microarray data from this study have been deposited in NCBI’s Gene Expression Omnibus under the accession number GSE50648.

### Statistical analysis

Raw microarray data was analyzed with Partek Genomics Suite software with probe intensities based on the median signal intensity of each feature and signal-to-noise ratio (SNR) data imported from GenePix Pro 6.0. GenePix Pro calculates SNR as the difference between median spot signal and median background divided by the standard deviation of the background signal. Data preprocessing comprised manual inspection of each extracted gene feature and quality control. We selected only unsaturated probes with an SNR greater than or equal to three (SNR ≥ 3) for analysis and performed quantile normalization across arrays to control for inter-array variability. Normalized probe intensities were then log transformed. We also removed probes without Ensembl annotation producing a subset of 15,818 probes which mapped to 7,909 genes (Additional file 1: Table S1). We performed three sets of ANOVAs using Partek Genomics Suite to identify probes that were differentially expressed between each treatment group and its respective control. Each set consisted of data from all the replicate pools of fish exposed to a specific metal and the replicate pools of unexposed fish housed in adjacent tanks during the metal exposure. The ANOVA model included terms for treatment (unexposed or exposed), concentration (control, low, mid, or high) and in interaction term for treatment*concentration. Contrasts were performed to determine significance between each concentration and control. We used a step-up Benjamini and Hochberg false discovery rate (FDR) of 0.01 to select differentially expressed probes. An FDR alpha value equal to 0.01 was chosen as the cut-off for the combined datasets of all replicate pools. Probes not meeting this threshold were filtered out and the resultant list was submitted to a second filter specifying a 1.8-fold-difference between treated vs. control samples. Only transcripts for which probes passed these filters were included in the final list (Additional file 1: Table S1). Fold changes for each probe pair (single probe transcripts excluded) were then averaged to generate a single value for each transcript.

Gene Ontology (GO) enrichment analysis was performed using the web-based tool GOTree Machine (GOTM; http://genereg.ornl.gov/gotm/), which generates a tree-like structure to navigate the GO Directed Acyclic Graph for input gene sets. GOTM supports analysis of the zebrafish genome; however, this analysis had to be performed at the gene rather than the transcript level. GO term and KEGG pathway (http://bioinfo.vanderbilt.edu/webgestalt/) enrichment analyses were then performed on the secondary lists to determine biological processes that are significantly (FDR = 0.1) enhanced or depressed by each metal. The enriched GO terms and KEGG pathways were also manually annotated into top level biological categories to clarify the overarching biological “themes” related to metal-induced gene perturbations. Finally, transcription factor enrichment was performed using MetaCore’s algorithm (GeneGo, Inc.) with settings enabled for identification of node relationships encompassing only direct downstream transcriptional regulation. Zebrafish genes were mapped to their human homologs using the Biomart feature in Ensembl with genes mapping one-to-many discarded from subsequent analysis. Background reference sets comprised the set of all transcripts with SNR ≥ 3 in each platform that could be subsequently mapped in a one-to-one fashion to their human homologs. Thresholds were set at an FDR = 0.1 with at least three DEG target identified for each enriched transcription factor.

Metacore does not explicitly provide zebrafish transcription factor regulatory networks; however, we anticipated that there would be high degree of conservation between the zebrafish and human networks [30], and mapped the zebrafish DEGs to their human orthologs before performing enrichment. Each differentially expressed transcript was mapped to its corresponding gene using the Ensembl database and resultant gene lists queried against their appropriate reference gene lists. Background reference sets comprised the set of all transcripts with SNR ≥ 3 in each exposure condition that could be subsequently mapped to Ensembl genes. Significantly enriched GO terms, or those GO terms that are statistically over-represented in each treatment compared to the reference set, were determined using the hypergeometric test with *p*-values adjusted using the Benjamini & Hochberg FDR correction (α = 0.1) and setting a threshold for the minimum number of genes per category (n = 3). While many statistical tests have been used for GO enrichment evaluation, the hypergeometric distribution provides an appropriate method for modeling data in which genes can be selected only once, i.e. sampling without replacement, as occurs in GO enrichment analysis [31].

## Results and discussion

### Gross changes, behavior, and histopathology

**Figure 1:**
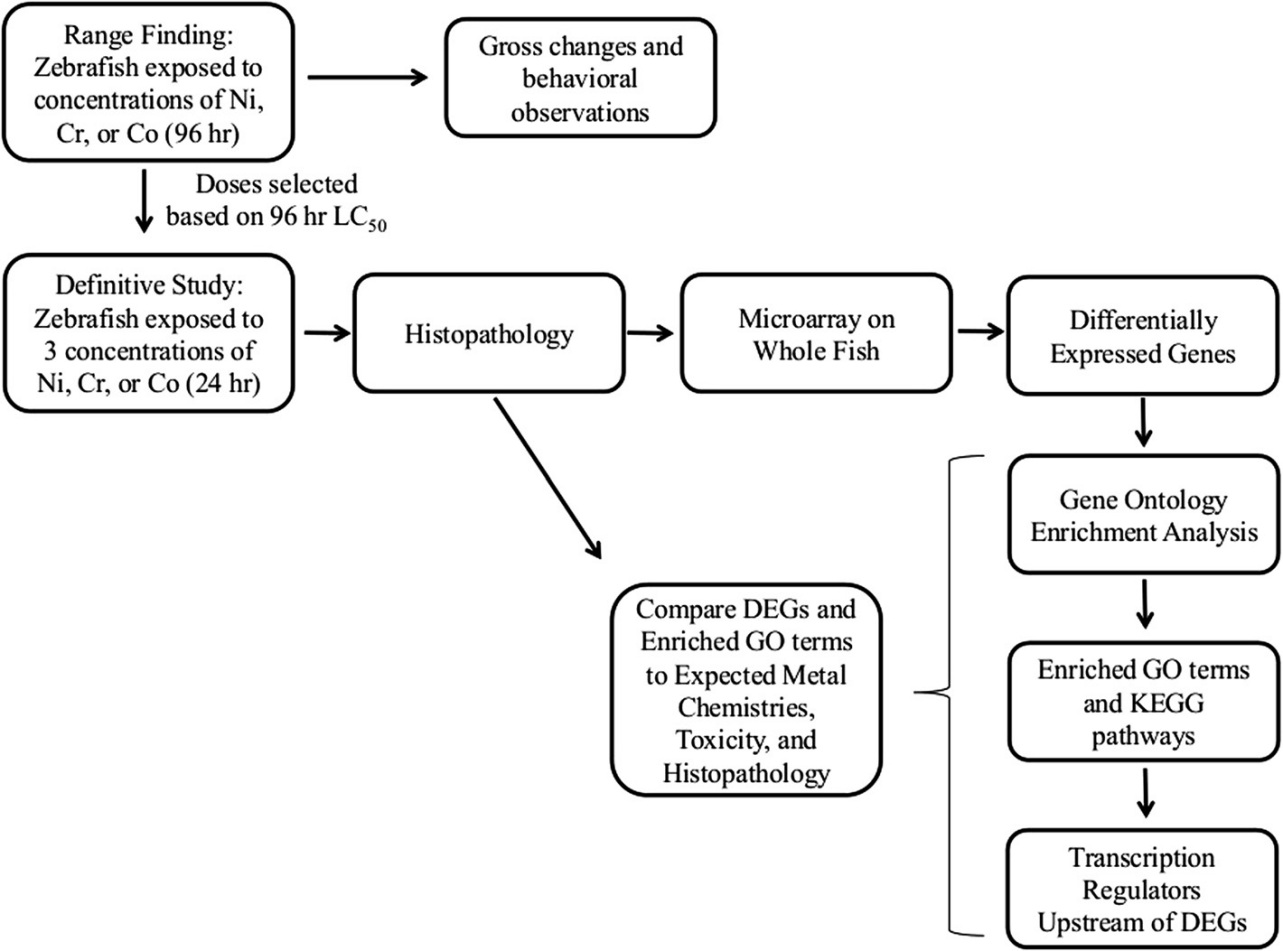
**Schematic of experimental design.** Diagram of the experimental design used for rangefinding and exposures.

Figure 1 provides a schematic of the experimental paradigm. During range-finding metal exposure studies, fish were qualitatively assessed for gross changes in behavior and general appearance. At study termination, tissues were stained with hematoxylin and eosin (H&E) to determine the morphological changes associated with each metal poisoning at the concentrations listed in Table 1.

Nickel-exposed fish appeared fuzzy, an appearance which is generally attributable to the excretion of mucus from goblet cells following irritation (personal communication, Dr. Donald K. Nichols). There were no deaths observed at any of the nickel concentrations nor did any of the nickel concentrations lead to any discernible histopathologic alterations (Table 2) although behavioral differences and skin abnormalities were qualitatively different between control and treated fish (i.e. sluggish swimming and fuzzy skin appearance). Previous studies indicate that gills, liver, and kidney are histopathological targets of nickel poisoning [22,23,32-34], but differences in fish species, exposure time and concentration could account for the discrepancy in the histopathological endpoints.

**Table 2:** Summary of histopathology

**Treatment**	**Morphological alterations**
NiCl_2_	No significant changes observed in any tissues examined.
CoCl_2_	Acute damage only to the olfactory organs including various inflammatory, degenerative, metaplastic, and necrotic lesions extending from the nasal cavity to the lungs. Intranasal lesions include olfactory epithelium necrosis (5 L, 5 M, 5H), lymphocytic inflammation (3 L, 4 M, 4 H), reactive hyperplasia (0 L, 0 M, 1 H), and olfactory lamellae fusion (0 L, 1 M, 3 H).
Na_2_Cr_2_O_7_	Acute damage only to the gills, intestine, and pharynx. Gills exhibited multifocal lesions consisting of lamellar fusion (4 L, 4 M, 5 H), epithelial hyperplasia (3 L, 4 M, 5 H), mononuclear cell infiltration (1 C, 4 L, 4 M, 5 H), epithelial necrosis (0 L, 4 M, 5 H), presence of thrombi in vessels (0 L, 4 M, 3 H) and hemorrhage (0 L, 2 M, 3 H). Intestine exhibits moderate to moderately severe atrophy of the mucosal folds (5 L, 5 M, 5 H), mild mononuclear infiltration of the lamina propria (5 L, 5 M, 5H) and mild necrosis of mucosal epithelium (2 L, 3 M, 3 H). Pharynx exhibits epithelial atrophy characterized by decreased mucosal thickness and loss of mucous secreting cells; also accompanied by mononuclear cell infiltration (0 L, 2 M, 4 H). Necrosis of pharyngeal epithelium occurred in 1 H.

Histopathological perturbations in zebrafish exposed to nickel, cobalt, or chromium for 24 hours. A total of five fish were examined per concentration including the control. In each case the numbers of instances of a finding are indicated in parentheses. C = control, L = low, M = mid and H = high concentration.

**Figure 2:**
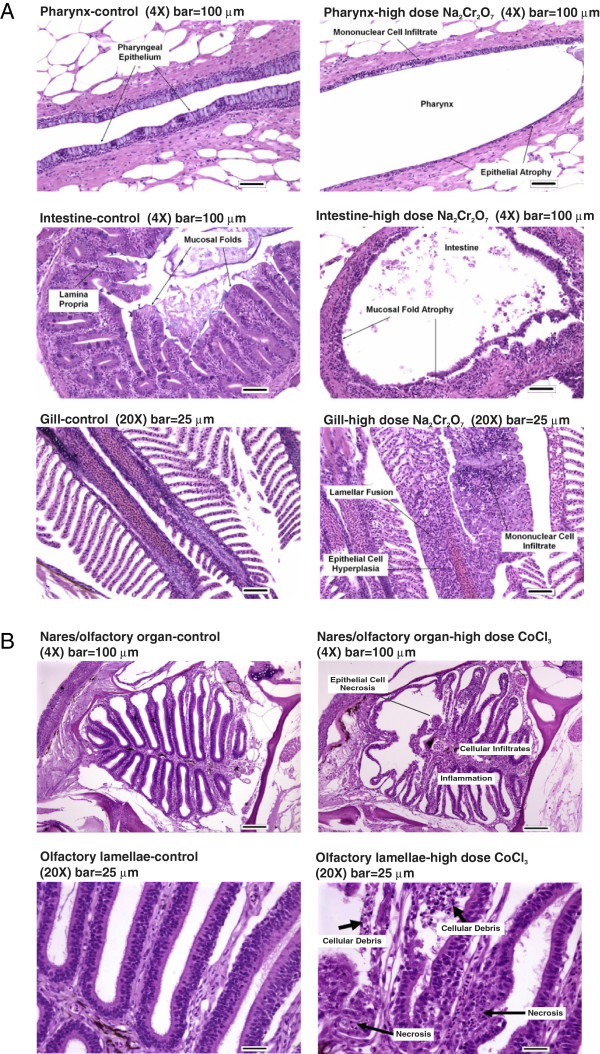
**Histopathology of affected tissues.** Histopathology of affected tissues after **(A)** chromium – pharynx, intestine, and gill or **(B)** cobalt – nares and olfactory lamellae – exposure compared to unexposed controls (left panels). Hematoxylin and eosin staining. Arrows indicate regions with metal-induced pathology.

Chromium poisoning caused visible changes in fish behavior and general appearance, including sluggish swimming, gasping and ulcerations near the tail in some fish. Four zebrafish died at the highest dose. Exposure to all three concentrations of chromium histopathologically affected the gills, intestine, and pharynx (see Table 2 and Figure 2A). Both gill and pharynx epithelium exhibited mononuclear cell infiltration, which is indicative of acute inflammation. The most prominent change in intestine was moderate to moderately severe atrophy of the mucosal folds and a mild infiltration of the intestinal lamina propria with mononuclear cells. Earlier studies report that the gills, kidney, and liver are histopathological targets of hexavalent chromium exposure in several species of freshwater fish [35,36]. The histopathology associated with this study supports the hypothesis that chromium exposure affects certain physiological processes including respiration, metabolic regulation, and possibly feeding.

During the cobalt range-finding studies, zebrafish exposed to high doses showed less schooling behavior, more surfacing, and less overall movement than the controls. In zebrafish, these behaviors are usually indicative of abnormal respiration and physiological stress. There were six deaths at the high dose and one death at the mid dose. Histopathology confirmed that zebrafish exposed to all three concentrations of cobalt presented with respiratory tract lesions specific to the olfactory epithelium (see Figure 2B and Table 2). The gill epithelium was unaffected, suggesting that the olfactory epithelial injury is specific to cobalt rather than a nonspecific reaction to waterborne irritants. Consistent with these results, inhalation studies in rats and mice confirm cobalt-specific morphological damage to the olfactory epithelia [37]. NOEL (no-observable-effect-level) could not be determined for any of the structural changes observed in fish treated with cobalt at the concentrations we tested.

### Transcriptomic responses to metal poisonings

**Figure 3:**
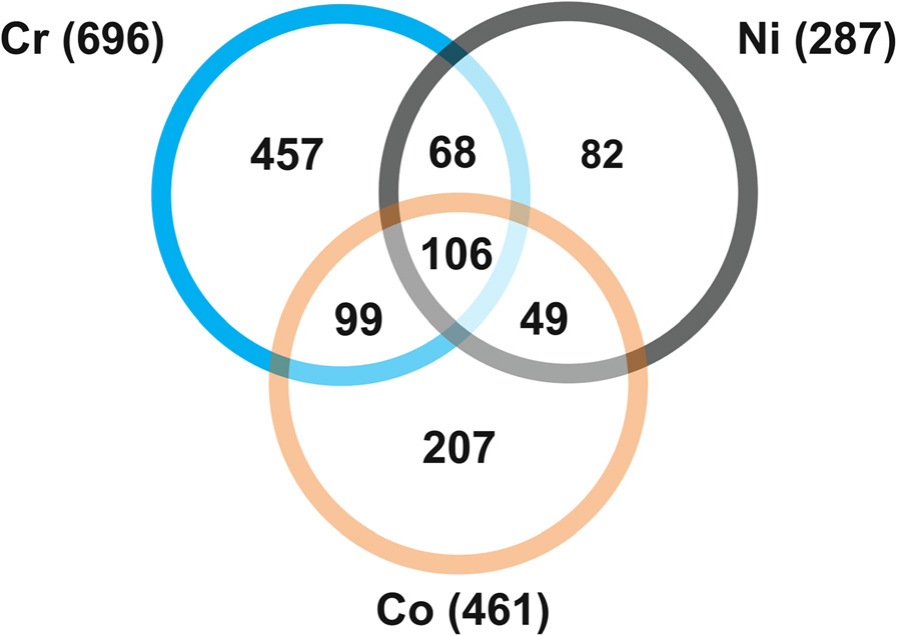
**Observed genes altered in expression.** Total number of observed genes significantly (FDR = 0.01) altered in expression by at least 1.8 fold in response to chromium (Cr), cobalt (Co), or nickel (Ni) versus untreated controls.

To identify differentially expressed genes (DEGs) resulting from nickel, cobalt, or chromium exposure, we compared the expression of genes in whole fish exposed to each metal with unexposed controls. Chromium, cobalt, or nickel exposure significantly altered expression of 696, 461, and 287 genes, respectively (Figure 3). There was a steep concentration-response relationship between mortality and metal concentration, with relatively little difference in the gene response to the three poisoning concentrations in the surviving fish as assessed by principal components analysis and analysis of variance (ANOVA). The concentrations were chosen to evoke histopathologic changes in an acute setting. Thus, the observed lack of an appreciative concentration-response curve would result if the doses fell in a nearly vertical region of the typical sigmoidal dose–response curve. Dose-dependent effects may have been lost in experimental variation. Lower doses or a longer time interval may be necessary to elicit a true dose–response curve.

### Biological processes perturbed by metal exposures

**Figure 4:**
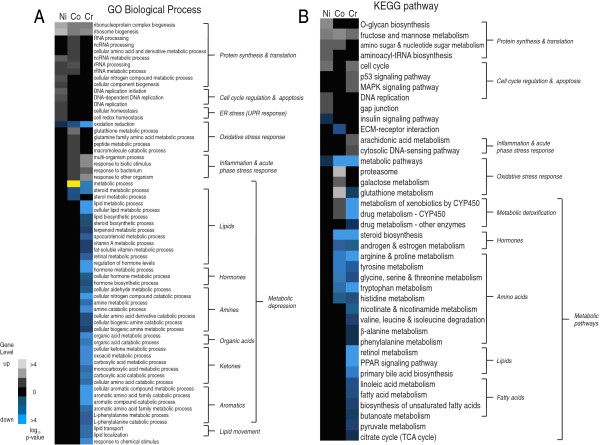
**Enrichment analyses. A** – Gene ontology term enrichment analysis for biological processes that are significantly (FDR = 0.1) enriched for up-regulated or down-regulated gene sets with at least 3 genes/transcripts present in a category to be considered significant by each metal. NOTE: The yellow highlight indicates that the enriched GO term “metabolic process” contained both up-regulated and down-regulated gene sets. **B** – KEGG pathway enrichment analysis for biological processes that are significantly (FDR = 0.1) enriched for up-regulated or down-regulated gene sets with at least 3 genes/transcripts present in a category to be considered significant by each metal. Metals listed on each heat map from left to right: nickel (Ni), cobalt (Co) and chromium (Cr) respectively. Color scale is log *p*-value of enrichment. White is for up-regulated gene sets and blue for down-regulated gene sets.

DEG lists were further subdivided into secondary lists by direction of response: for example, of the 287 genes induced by nickel, 97 were down-regulated and 190 were up-regulated. GO term enrichment analysis and KEGG pathway enrichment analysis inferred biological processes modified by each metal. The biological processes identified in the gene ontology enrichment analyses (GO biological processes and KEGG pathways) fell into five categories with differential responses for the three metals: protein synthesis and translation; altered reduction-oxidation (redox) levels; inflammation and acute phase stress response; cell cycle regulation and apoptosis; and metabolic depression (Figure 4 and Table 3). Table 3 summarizes the biological processes, chemistries and toxicities and results of pathway enrichment analysis for metals compared to histopathologic findings. Biological processes identified in the transcriptomic analysis were compared at the pathway level to published mechanisms of aquatic toxicology. In general, metals affect the cellular heme content, impairing oxidative function of cells [13,38-40]. Consistent with this observation, all three metals showed down-regulation in biological processes associated with the oxidative stress response, including oxidation-reduction (Figure 4A) and metabolic pathways in the oxidative stress response (Figure 4B). Further, all three metals induced genes in GO biological processes (most notably ribosome biogenesis) and KEGG pathways regulating protein synthesis and translation (Figure 4A and B). Increased demand for newly synthesized proteins may result from enhanced requirements for translation of stress responsive genes particularly those involved in combating oxidative and inflammatory stress. It is also possible that the enhanced protein synthesis is a compensatory mechanism to replenish cells lost through apoptosis in tissues specifically targeted by metal exposures. The literature is conflicting regarding ribosome biogenesis in response to toxicant poisoning [41-46]. The discrepancies in our study between metals may reflect differences in the severity of toxic insult among the metals.

**Table 3:** Comparison of histopathology, chemistry, toxicity and observed gene response among metals

**Metal**	**Predicted**	**Histopathology target**	**GO biological process**	**KEGG pathway**	**Genes**	**References**
All (Ni, Co, Cr)	Effects on heme carriers (respiration)	n/a	Cell cycle regulation and apoptosis	Cell cycle regulation and apoptosis	*mcm4, gadd45bl, orc6l*	[57]
Ni	Oxidative damage to DNA	None	Oxidative stress response	Oxidative stress response	*Periredoxin (zgc:110343), thioredoxin (zgc:92903); txnl1; pdia4; pdia4; pdip5; hif1a; xbp1; abcf2; pfkfb3; il1b; egln3; hk2; hspa5; pdia4, dnajb11, dnaja4, jnajb11, ahsa11, hsp70l, hspe1, hsp90b1, hspa4l*	[61–66. 70–91]
			Endoplasmic reticulum (ER) stress and UPR (unfolded protein response)	n/a	*dnaja4, jnajb11, ahsa11, hsp70l, hspe1, hsp90b1, hspa4l; hspa5; pdia4; dnajb11*	[61-69]
			Protein synthesis and translation (DNA replication processes)	Protein synthesis and translation	*mcm4, orc6l; mcm3, mcm5; rbb4*	[57-60]
Co	Inducer of hypoxic response	*Olfactory organs*: inflammatory, degenerative, metaplastic, necrotic lesions	Oxidative stress response	Oxidative stress response	*gss (zgc:101574); gclc; zgc:110010; psmd3, psmd7, psma6b, psma5, psmc3, psmc4, psmd11b, psmd1, psmc6, psme3, psmc1b*	[40,72-74]
			Inflammation and acute phase stress response	Increased metabolic detoxification	Protein serum amyloid A (*zgc:103580* )*; il1b; c/ebp; atf3*	[75-78]
Cr	Oxidizing agent; induces oxidative stress and reactive oxygen species	*Gills*: hyperplasia, necrosis, hemorrhage, inflammation	Inflammation and acute phase stress response	Inflammation and acute phase stress response	*pcna, nfkbiaa, hamp1, ptgs2a, ptgs2b, ptges; dusp2; dusp5; dusp1; gadd45g; hmox1; socs1; egr2; hpx; il1b; c/ebp; atf3*	[52,53,75-78]
		*Intestine*: atrophy of mucosal folds, necrosis, inflammation	Ribosome biogenesis	Cell cycle regulation and apoptosis	*ccne2, rrm2, zgc:77806; pcna; stat3, p53*	[89-91]
		*Pharynx*: atrophy, mononuclear cell infiltration	Metabolic depression	Metabolic pathways	*g6pca, gys2, fabp1a, abcc2; hnf3 α, hnf3 β, hnf1 β, tcf8, bmal, ppargc1, lxr* α*, coup-tfi, gata-6*	[80-82,84-88]

Summary of gene responses consistent with histopathology and predicted chemistry and toxicity of metals after 24 hr exposure.

Nickel poisoning causes oxidative damage to DNA and inhibits antioxidant defenses [47-49]. Nickel-poisoned fish had less observable histopathology than the other metals and no histopathology directly attributable to nickel poisoning, but gene expression was more enriched in processes involving cell cycle regulation and apoptosis (Figure 4A) and protein synthesis and translation than the other metals (Figure 4A and B). The lack of histopathological signs of nickel poisoning at 24 hr may suggest that our nickel exposure scheme captures mechanisms which drive the initial response to metal poisoning (i.e. at 24 hr) prior to histologic alterations.

Cobalt poisoning mimics hypoxia, stimulating the production of reactive oxygen species and increasing lipid peroxides [50]. Consistent with this observation, cobalt showed more up-regulation of genes regulating the biological processes of the oxidative stress response than any other metal (Figures 4A and B).

Chromium redox cycling and ROS generation induce DNA damage and activate subsequent repair mechanisms [51,52]. Corroborating these mechanisms, chromium up-regulated p53 and MAPK signaling pathways (Figure 4B) [53]. Genes associated with inflammation and acute phase stress responses were up-regulated more with chromium than the other metals (Figure 4A and B). These results are consistent with the histopathology observations that chromium exposure induced the most inflammatory cell infiltration (Figure 2 and Table 2). The genes in pathways associated with metabolic depression support a molecular mechanism underlying the reduced feeding behavior and intestinal abnormalities observed in chromium exposed fish (Figure 2). Chromium poisoning was associated with a marked down-regulation in genes involved in cellular metabolism, including lipid and steroid metabolic pathways. These processes included the citrate (TCA) cycle and fatty acid metabolism, which are regulated by degradation of amino acids into acetyl-CoA and proprionyl-CoA intermediates respectively [54]. Genes controlling the biological and molecular processes controlling the processing of pyruvate, a degradation product of glucogenic amino acids, also significantly decreased in fish exposed to chromium (Figure 4A). These results suggest that chromium poisoning, but not cobalt or nickel, caused significant reductions in metabolic capacity, particularly for amino acids and processes associated with their metabolic by-products. These results are consistent with the observed intestinal mucosa atrophy and mild necrosis (Figure 2A). Chromium is readily absorbed by the intestinal tissue. Since gut mucosa represents the primary site for whole-body amino acid metabolism [55,56], mucosal atrophy can significantly diminish the gut’s amino acid metabolic capacity and decrease amino acid requirements and use.

### Enriched transcription factors in response to metal poisoning

**Figure 5:**
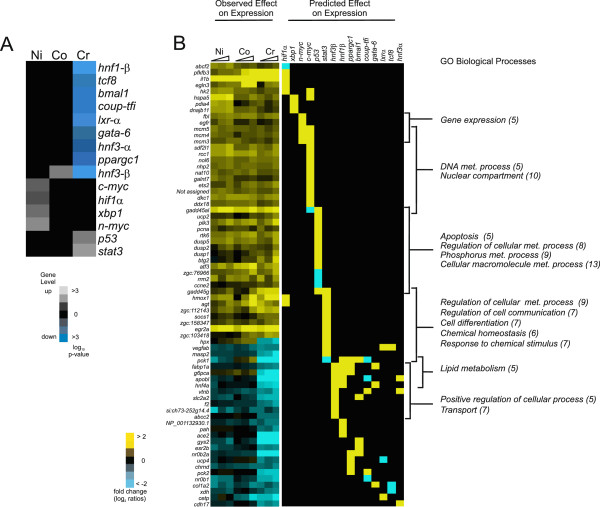
**Enriched transcription factors and predicted gene expression direction. A**- Statistically enriched transcription factors that modulate gene expression for both enhanced and repressed gene responses to metal exposures. **B** - Predicted direction of gene expression regulation (i.e. gene activation or repression) derived from the MetaCore knowledge base for human orthologs.

We performed transcription factor (TF) enrichment analysis using Metacore on each secondary enhanced/repressed gene list in an attempt to identify upstream regulatory networks that mediate metal induced gene expression linked to GO biological processes (Figure 5, Table 3, and Additional file 1: Table S1). The metals showed a significant discrepancy in the type and direction of the regulation of the transcription factors expressed (Figure 5A). Common to all metals was the up-regulation of the highly conserved mini-chromosome maintenance 4 (*mcm4*) gene with DNA helicase activity essential for the inhibition of eukaryotic genome replication and the origin recognition complex (*orc61*) which facilitates replication [57-60] (Figure 5B, Table 3, and Additional file 1: Table S1).

In nickel poisoning, transcripts for the DNA replication processes genes *mcm3*, *mcm5*, and *rbb4* were up-regulated in addition to *mcm4* and *orc61* (Table 3, Figure 5B, and Additional file 1: Table S1). Nickel induced transcription factor changes consistent with redox signaling, including up-regulation of *hif1α* and *xbp1* (Figure 5A and Table 3) and their associated gene targets (Figure 5B and Table 3). These gene products are cellular regulators that sense oxygen status and trigger adaptive cascades to maintain normoxia [61-63]. It is known that nickel stabilizes Hif-1α by preventing the degradation of the protein either through the depletion of ascorbate or by replacing iron in the hydroxylases responsible for Hif-1α degradation [64-66], leading to transcriptional activation of downstream targets. In our study, nickel poisoning up-regulated other genes critical for redox sensing and homeostasis, including periredoxin (*zgc:110343*), thioredoxin (*zgc:92903*), thioredoxin-like 1 (*txnl1*), and protein disulfide isomerase 4 and 5 (*pdia4*, *pdip5*) (Additional file 1: Table S1 and Figure 5B). Levels of *hif1α* downstream targets (*abcf2*, *pfkfb3*, *il1b*, *egln3*, *hk2*) were significantly up-regulated. *xbp1* expression was enhanced 1.52 fold in parallel with robust up-regulation of its gene targets (*hspa5*, *pdia4*, *dnajb11*) (Figure 5A and B). The *xpb1* gene target *hspa5* (which encodes Grp78/BiP) also represents a specific and key marker for induction of the unfolded protein response (UPR) [67-69], an adaptive response that prevents protein aggregation by enhancing expression of molecular chaperones and diminishing nascent polypeptide flux into the ER [70,71]. Nickel dose-dependently increased expression of *hspa5* (Figure 5B). Surprisingly, nickel poisoning did not induce genes involved in clearing terminally misfolded proteins (i.e. members of the ER-associated protein degradation machinery [ERAD] family) (Additional file 1: Table S1 and Figure 5B) [71]. However, nickel poisonings up-regulated an additional ensemble of ER chaperones (*dnaja4*, *jnajb11*, *ahsa11*, *hsp701*, *hspe1*, *hsp90b1*, *hspa41*) which play a crucial role in ensuring proper protein folding. Taken together, our data suggest that nickel exposures induced the UPR via *xbp1* transcriptional activation.

Cobalt poisoning resulted in up-regulation of only *hnf3β* in the Metacore analysis (Figure 5A). Genes were up-regulated in two predominant biological processes: (1) altered redox levels (e.g., the glutathione synthesis genes [*gss (zgc:101574)*, *gclc, and zgc:110010*] (Additional file 1: Table S1)*,* and genes which encode multiple structural components of both the catalytic (20S) and regulatory (19S) ribosomal subunits [*psmd3, psmd7, psma6b, psma5, psmc3, psmc4, psmd11b, psmd1, psmc6, psme3, psmc1b*]) [40,72-74] (Additional file 1: Table S1) and (2) inflammation and acute phase stress response (genes encoding protein serum amyloid A [*zgc:103580*], the pro-inflammatory cytokine *il1b*, *atf3*, *c/epb*) [75,76] (Table 3, Additional file 1: Table S1, and Figure 5B). The negative regulator *atf3* dampens the inflammation response by antagonizing the pro-inflammatory factor NF-κB, while *c/ebp* is robustly up-regulated by pro-inflammatory signals, including *il1b*, and functions as an enhancer of the inflammatory response [57,75-79].

The transcription factor analysis in chromium poisoning is consistent with the chromium-induced metabolic depression reported in the gene ontology analysis (Figures 4 and 5A). Chromium poisoning caused down-regulation of an entire set of genes encoding important regulators of energy metabolism, glucose, cholesterol, amino acid, and fatty acid metabolism and transport in many tissues, including the intestine and liver, including *hnf3α*, *hnf3β*, and *hnf1β*[80] (Figure 5A). *hnf4α* is a key transcriptional target of both *hnf3α* and *gata-6*[81,82] which represents a master transcriptional activator of energy metabolism genes in multiple tissue types [83]. Orphan nuclear receptors with putative roles in gut and liver metabolism of nucleic acids (e.g., *tcf8*; Figure 5A), carbohydrates and lipids (*bmal1, ppargc1*; Figure 5A), and sterol and steroid hormones (*lxr-α, coup-tfi*; Figure 5A) were also down-regulated [84-87] (Figure 5A). Genes including *g6pca*, *gys2*, *fabp1a*, and *abcc2* (involved in glucose metabolism, lipid metabolism, and canalicular bile acid transport, respectively) (Figure 5B and Additional file 1: Table S1) [88]. Inflammation and acute phase response genes were, for the most part, up-regulated by chromium, including pro-inflammatory genes (*pcna*, *nfkbiaa*, *hamp2*, *ptgs2a*, *ptgs2b*, *ptges*) (Figure 5B and Additional file 1: Table S1) and genes encoding multiple dual-specificity phosphatases which modulate inflammatory MAPK cascades (*dusp2*, *dusp5*, *dusp1*) (Figure 5B and Additional file 1: Table S1). Similar to nickel, chromium also up-regulated *il1b, c/ebp,* and *atf3* (Figure 5B and Additional file 1: Table S1). Chromium also up-regulated cell cycle regulation and apoptosis genes not enhanced in the other metals, including genes involved in the G_1_/S and G_2_/M cell-cycle checkpoints (*ccne2*, *rrm2*; Additional file 1: Table S1 and Figure 5B; [89]) and in the RAD6-dependent DNA repair pathway (*pcna*) [90,91] (Figure 5B). The transcription factor p53 was also up-regulated (Figure 5A), which mediates expression of protective genes that repair damaged DNA, power the immune system, arrest the proliferation of damaged cells, and induce apoptosis [92], as well as guards the cell-cycle checkpoint by inducing apoptosis under conditions of excessive oxidative stress and DNA damage [93].

The transcriptomic results for chromium in particular are consistent with the observed intestinal mucosa atrophy and mild necrosis observed in the histopathology (Figure 2A). Chromium is readily absorbed by the intestinal tissue. Since gut mucosa represents the primary site for whole-body amino acid metabolism [55,56], mucosal atrophy can significantly diminish the gut’s amino acid metabolic capacity and decrease amino acid requirements and use. This hypothesis is supported by the observed down-regulation of liver-specific genes after chromium exposure. Robust, differential down-regulation of key mediators of glucose metabolism (*g6pca, gys2*), lipid metabolism (*fabp1*), and canalicular bile acid transport (*abcc2*) (Figure 5B) suggest metabolic perturbations in the liver and/or gut-liver axis [88]. Taken together, the biological processes, transcription factors and histopathology data may suggest modulation of intestinal metabolic pathways due to chromium exposure. It is unclear whether this is a direct consequence of chromium poisoning or a secondary consequence of chromium-mediated decreases in feeding over the 24 hr exposure period. A previous study in freshwater trout exposed to nonlethal hexavalent chromium (1/10 of the 24 hr LC_50_) reported intestinal atrophy and decreased brush border enzymatic activities consistent with decreased feeding behavior [94]. However, since animal care personnel did not observe changes in feeding behavior, it is more likely that chromium mediates specific perturbations to the gut microenvironment that ultimately trigger metabolic depression.

### Limitations

The technical approaches used in this work have both strengths and weaknesses. In using whole adult organism RNA preparations, we introduce the ability to detect toxicity responses at the whole organism level. We did obtain gene expression level data that corroborates the histological observations in some cases (e.g., chromium-induced gut histopathology), but we acknowledge that the detection of clear organ-specific effects was not possible with this approach because of the dilution of expression signals for genes that have highly tissue-specific distributions. Highly similar gene expression profiles are probably shared by multiple tissues, making it difficult to differentiate among toxicity signatures for individual target organs from the system-wide response.

Although we measured tank levels of each respective metal, we acknowledge that this does not necessarily indicate bioavailable dose, and that the comparison across studies would require internal measurement of metal concentration within the organism. The lack of a dose–response relationship in histopathology is a significant limitation to this study. The high doses required to observe histopathologic changes in the acute time interval of the study precluded identification of subtle, dose-dependent and tissue-specific responses. It is striking that despite establishing an exposure regimen from a 96 hr mortality curve, there were few differences in gene responses across the exposure levels, even though we tested gene responses 48 hr earlier than the mortality curve end points. This effect could be the result of selecting doses in a nearly vertical region of the typically sigmoidal dose–response curve with little difference between the LC_20_ and LC_60_ doses. Lower doses or longer time intervals could better delineate the difference in histopathologic response in future experiments.

This observation also suggests that range-finding for mechanistic toxicity studies such as this one should not solely be based on mortality data. Some method of establishing dose responsiveness based on gene expression or other molecular endpoints are necessary as well.

Nonetheless, the present study supports the use of transcriptomics in the intact organism to predict candidate genes associated with toxicity endpoints in response to an external chemical insult. Using this approach, we have identified novel gene and transcription factor targets that mediate the response to metal toxicity. Finally, these new players provide hypothesis-generating targets for future evaluation in classically designed studies of the mechanisms of heavy metal toxicity.

## Conclusion

We developed an exposure paradigm for comparing the effects of various metals with varying toxicity mechanisms of action using whole animal transcriptomics in the zebrafish vertebrate model. Using this technique, we identified changes in expression of groups of genes consistent with adaptive responses to toxicity induced by nickel, cobalt, or chromium, including acute phase response, cell cycle regulation, apoptosis, and metabolic depression, among others. Histopathological evaluations corroborate the toxicity endpoints derived from gene-level based pathway analysis for chromium and cobalt.

Many of the genes enriched for biological processes’ gene responses reported in this study are consistent with known physiological endpoints in there metals. Nickel induces oxidative damage to DNA and proteins; genes were up-regulated for biological processes including protein synthesis and translation, and cell-cycle regulation and apoptosis. Cobalt induces hypoxia; genes regulating biological processes of redox response, protein synthesis and translation, and inflammation and acute phase stress response were up-regulated. Chromate is a strong oxidizing agent and damages DNA integrity; biomolecular pathways and genes associated with inflammation and acute phase stress response were up-regulated and gene signatures suggested metabolic depression occurred. Further, a number of novel transcription factors that mediate gene induction at the transcriptional level in response to metal exposures were identified.

Enrichment of several functional categories of genes plausibly involved in a variety of biological responses was identified using unsupervised gene ontology analysis of metal-specific gene responses. Unique histopathological alterations were identified for each metal exposure, consistent with metal-specific toxicity in target organs and tissues. For nickel, we find that our toxicogenomic approach using whole organism RNA preparations may be more sensitive for identifying targets of nickel toxicity than the classic toxicology approach of histopathology. These results suggest that toxicogenomics in the whole adult zebrafish may provide a robust model for identifying leading indicators of toxicity and intervention approaches for exposure to toxic chemicals.

Using a transcriptomics approach, we identify a number of upstream modulators of metal-induced gene expression. A number of these transcription factors have been previously implicated in triggering metal-specific gene responses to toxicity (*p53, Hif1a*). Multiple novel mediators of the toxic response to nickel, cobalt, or chromium in whole adult zebrafish, including *Xbp1*, various *Hnf* members, and *Gata6* were also identified. These findings provide additional mechanistic information on metal toxicity mechanisms and highlight novel potential points of intervention for treatment of metal poisoning.

## Competing interests

The authors declare that they have no competing interests.

## Authors’ contributions

Conceived and designed the experiments: DAJ, JAL. Performed zebrafish exposures, sample processing and microarray analysis: CEB. Analyzed the data: NH, JAL, DAJ. Wrote the paper: NH, JAL, DAJ, CEB, DLI, JDS. All authors read and approved the final manuscript.

## Pre-publication history

The pre-publication history for this paper can be accessed here:

http://www.biomedcentral.com/2050-6511/15/15/prepub

## Supplementary Material

Additional file 1 **Final gene set.** Final gene set used for analysis – 7,909 genes with 3 additional tabs each one containing a list of differentially expressed genes (DEG) per chemical with fold change data. A gene was considered DE if it was significantly (FDR = 0.01) altered in expression by at least 1.8 fold in response to chromium (Cr), cobalt (Co), or nickel (Ni) versus untreated controls. Microsoft Excel workbook. Click here for file

## Disclaimer

Opinions, interpretations, conclusions, and recommendations are those of the authors and are not necessarily endorsed by the U.S. Army. Research was conducted in compliance with the Animal Welfare Act, and other Federal statutes and regulations relating to animals and experiments involving animals and adheres to principles stated in the Guide for the Care and Use of Laboratory Animals (NRC 2011) in facilities that are fully accredited by the Association for the Assessment and Accreditation of Laboratory Animal Care, International. The research described herein was sponsored by the U.S. Army Medical Research and Materiel Command, Military Operational Medicine Research Program. Citations of commercial organizations or trade names in this report do not constitute an official Department of the Army endorsement or approval of the products or services of these organizations.
